# Dichorionic-Diamniotic Twin Pregnancy in a Bicornuate Uterus With Twin A Affected by Ebstein’s Anomaly

**DOI:** 10.7759/cureus.38248

**Published:** 2023-04-28

**Authors:** Colleen N Gorman, Spencer A Grabois, Shreya Mathur, Evan P Grabois, Allen Silanee

**Affiliations:** 1 Obstetrics and Gynecology, Nova Southeastern University Dr. Kiran C. Patel College of Osteopathic Medicine, Davie, USA; 2 Obstetrics and Gynecology, Mount Sinai Medical Center, Miami, USA; 3 Pediatrics, Jackson Memorial Hospital, Miami, USA

**Keywords:** mullerian anomaly, renal agenesis, congenital malformations, ebstein’s anomaly, bicornuate uterus

## Abstract

Congenital Mullerian anomalies are rare developmental defects that result in malformation of the fallopian tubes, uterus, cervix, and vagina. The bicornuate uterus is one of the many variants of Mullerian anomalies, defined as having an external fundal indentation of greater than one centimeter. Pelvic ultrasound has a sensitivity of 99% for identifying bicornuate uteruses and is the predominant imaging device used for diagnosis. The cervical and uterine cavity anatomy in patients with a bicornuate uterus varies. The effect of maternal uterine structure on offspring development has been poorly documented. This report details a rare case of dichorionic-diamniotic twin pregnancy in a bicornuate uterus with one fetus affected by Ebstein’s anomaly. Twin A was diagnosed by first-trimester ultrasound with right renal agenesis and Ebstein’s anomaly. Twin B did not have any anatomical defects identified on ultrasound. Both twins were delivered via emergency repeat cesarean section at 34 weeks and four days due to nonreassuring fetal heart tracings and twin A in the breech presentation. Twin A and twin B were found to be in separate horns within the uterus during low transverse cesarean section. Twin A required endotracheal intubation in the delivery room due to respiratory distress. Both twins required neonatal intensive care treatment. Twin A was found to have a right pelvic kidney, rather than right renal agenesis, while in the neonatal intensive care unit. Females with germline mutations in the Mullerian duct and urogenital sinus development have resulted in concomitant malformations in the uterus and kidneys. This is a rare case of an infant with a cardiac anomaly born to a mother with a germline mutation. The relationship between congenital heart defects and uterine anomalies has not been identified. As seen in this case, maternal malformations impacting fetal cardiac development can be sporadic or result from germline mutations in mesoderm that have not been reported yet.

## Introduction

Ebstein’s anomaly

Ebstein’s anomaly is a rare congenital malformation of the tricuspid valve (TV) and right ventricle. The condition is characterized by apical displacement of the posterior and septal TV leaflets and atrialization of the right ventricle. Ebstein’s anomaly has a prevalence of 0.3-0.6% of all congenital heart defects. Most cases of Ebstein’s anomaly are idiopathic, although some studies have suggested familial inheritance [1]. Isolated genetic defects involving chromosomes 15q, 11q, myosin heavy chain 7, and NKX2.5 have also been associated with Ebstein’s anomaly [1]. Maternal exposure to lithium, benzodiazepines, and varnishings has also been noted in association with Ebstein’s anomaly [1].

The severity and clinical presentation of Ebstein’s anomaly vary. Mild apical displacement of the TV is often asymptomatic in adulthood, whereas moderate TV displacement presents as cyanosis or heart failure in infancy or childhood. With severe TV displacement, there is a risk of in-utero heart failure, resulting in nonimmune hydrops fetalis and death. The clinical findings of Ebstein’s anomaly in a neonate or child may include cyanosis, cardiomegaly, heart failure, and respiratory distress [2]. There is an increased risk of early death from heart failure and pulmonary hypoplasia in fetuses, neonates, and infants. However, individuals with Ebstein’s anomaly and isolated cyanosis usually demonstrate a spontaneous improvement in cyanosis as pulmonary vascular resistance decreases [3]. Ebstien’s anomaly may present in adolescence and adulthood with arrhythmias, exertional dyspnea, fatigue, palpitations, or sudden cardiac death [2].

Intrauterine diagnosis of Ebstien’s anomaly is primarily made with a fetal echocardiogram showing an enlarged right heart and tricuspid regurgitation. The septal leaflet of the TV being displaced more than 8 mm/m^2^ from the cardiac crux and the elongation of the anterior leaflet of the TV is suggestive of Ebstein’s anomaly [4]. Cardiomegaly and right atrial enlargement can be identified on neonatal chest x-ray, with a cardiothoracic ratio greater than 0.65 being associated with poor outcomes. Cardiac MRI can complement echocardiograms in patients affected by Ebstein’s anomaly, allowing for better visualization and functional assessment of the right ventricle [4]. 

Initial postnatal management involves stabilization of cardiorespiratory status with potential intubation, mechanical ventilation, prostaglandin infusion, inhaled nitric oxide, and inotropic support [4]. The prognosis of Ebstien’s anomaly varies based on the severity of the disease, with mortality in the first year of life being approximately 30%.

Bicornuate uterus

Beginning in the seventh week of embryological development in female fetuses, degeneration of Wolffian ducts and differentiation of Mullerian ducts will give rise to the uterus, cervix, fallopian tubes, and upper third of the vagina [5]. The uterus is formed by the fusion of the Mullerian ducts in the midline during the eighth week of gestation. Fusion of the Mullerian ducts results in the Mullerian tubercle, which interacts with the urogenital sinus and helps form the vaginal canal. Uterine anomalies can result from the failed fusion of the Mullerian ducts, one of them being a bicornuate uterus. The cause of the bicornuate uterus is thought to be multifactorial, with genetic and environmental influences. 

Proper Mullerian duct development depends on several genes, such as Pax2, Fgf, Ctnnb1, Bmp, and Wnt signaling molecules and homeodomain transcription factors [6]. Disruptions in these molecular pathways alter the uterine anatomy and create a potentially inhospitable environment for future fetuses. Environmental exposure to agents such as diethylstilbestrol and thalidomide in utero has led to various uterine anomalies, with the severity dependent on the onset and duration of their use during the pregnancy [7]. 

Diagnosis of a bicornuate uterus typically does not happen until pregnancy occurs. Dysmenorrhea and menorrhagia from having two uterine cavities can prompt women to present during adolescence. There are significant reproductive and obstetric complications associated with the bicornuate uterus. While the presence of a bicornuate uterus in the general population is 0.4%, it is responsible for 2.1% of miscarriages and 4.7% of female infertility. The risk of preterm delivery, pregnancy loss, intrauterine growth restriction, and malpresentation at delivery are significantly higher in women with bicornuate uterus [8]. 

The association between congenital malformations and bicornuate uterus has been reported. The association of disrupted cardiac development in fetuses to mothers with bicornuate uteruses has not been well documented. We present an extremely rare case of Epstein’s anomaly and right pelvic kidney in one fetus of a dichorionic-diamniotic twin pregnancy in a patient with a bicornuate uterus. Genetic disorders in mothers can be inherited by offspring, and germline mutations involving renal and uterine development have been associated in the past. In this specific case, cardiac structures were also affected. This report emphasizes the role of embryonic signaling pathways and genes responsible for embryogenesis on fetal structure development, with unidentified mesoderm mutations as the potential cause of the congenital malformations identified in twin A. 

## Case presentation

Patient history 

A 37-year-old female G2P1 presented to the hospital with the onset of labor at 34 weeks and four days gestation with dichorionic-diamniotic twins. She received routine prenatal care in El Salvador before continuing prenatal care in the United States at 32 weeks gestation. She reported no past medical history or allergies. Her medications included prenatal vitamins, iron, and vitamin C. She denied alcohol, tobacco, and recreational drug use. Surgical history included a cesarean section. She denied any family history, including congenital anomalies or multiple gestations.

Obstetrical history

**Figure 1:**
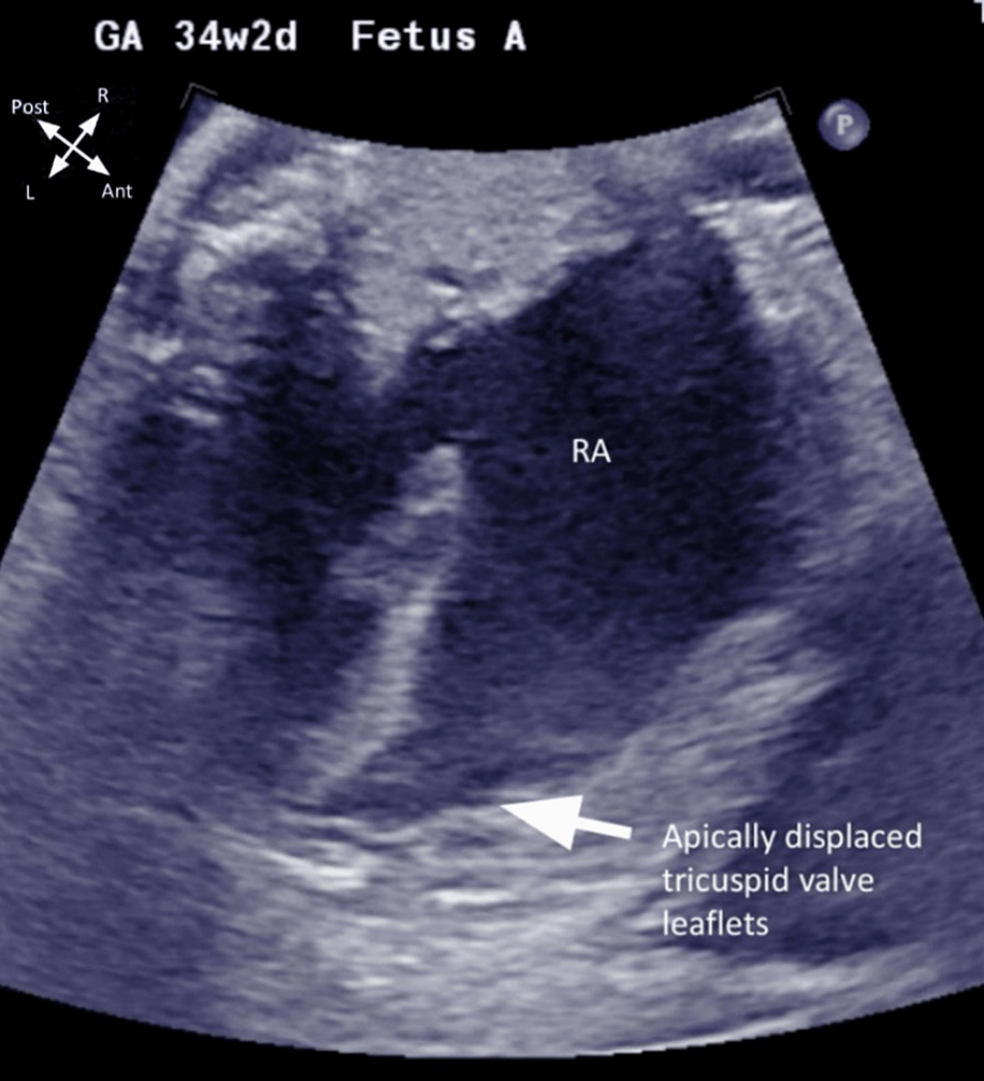
Fetal echocardiogram showing apical four-chamber view of Twin A with Ebstein's anomaly RA, right atrium

The patient’s obstetrical history includes a prior singleton pregnancy in 2018 complicated by intrauterine growth restriction (IUGR). She was delivered via cesarean section at 38 weeks for this reason, with no anatomical anomalies identified in the offspring. Her current twin pregnancy is complicated by a bicornuate uterus. In El Salvador, she had a prenatal diagnosis, confirmed by ultrasound, of right renal agenesis and Ebstein’s anomaly in twin A. Her ultrasound at 31 weeks gestation showed twin A measuring 1215 grams, breech presentation, posterior placenta, and an amniotic fluid index (AFI) within normal limits. Twin B measured 1353 grams, cephalic presentation, posterior placenta, AFI within normal limits, and no anatomical defects. Fetal echocardiogram at 34 weeks and two days gestation confirmed twin A’s diagnosis of Ebstein’s anomaly (Figure 1).

Labor and delivery course

**Figure 2:**
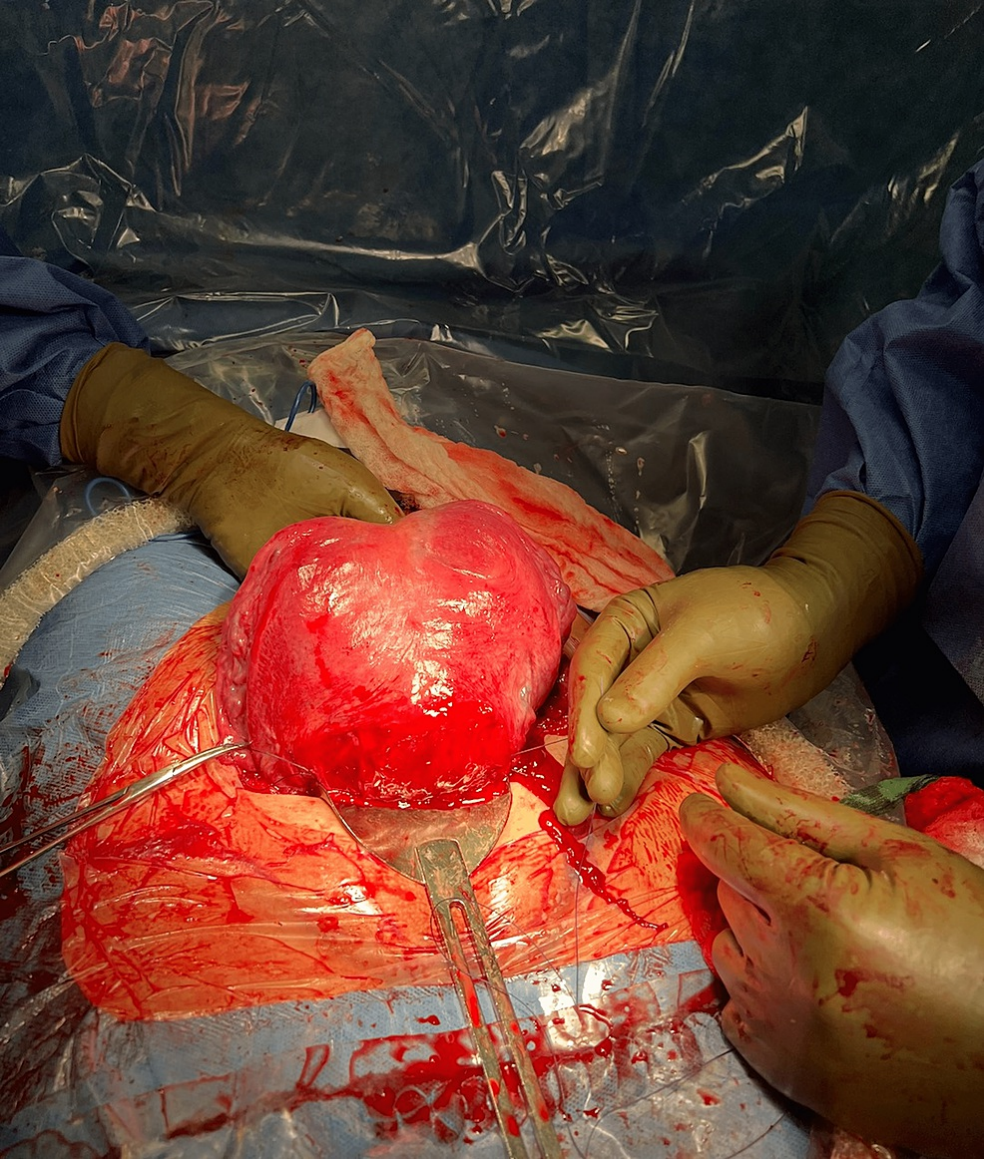
Bicornuate uterus during hysterotomy repair

The patient presented to the hospital with contractions occurring every 10 minutes. Persistent category two fetal heart tracing was detected in twin A, along with a breech presentation, requiring an emergency cesarean section to be performed. During the delivery, twin A was noted to have a nuchal cord that was reduced. Two viable female infants were delivered atraumatically. The placenta of twin A was 50% abrupted, and the placenta of twin B was intact. Externalization of the uterus during hysterotomy repair allowed for visualization of the bicornuate uterus (Figure 2).

Twin A’s Apgar scores were six, eight, and eight at one, five, and 10 minutes respectively. Twin A demonstrated signs of respiratory distress, which required endotracheal intubation in the delivery room. Twin B’s Apgar scores were nine and nine at one and five minutes, respectively. Twin A had a birth weight of 1602 grams, and Twin B weighed 1787 grams. Both of the neonates were transferred to the neonatal intensive care unit.

Neonatal intensive care unit course 

**Figure 3:**
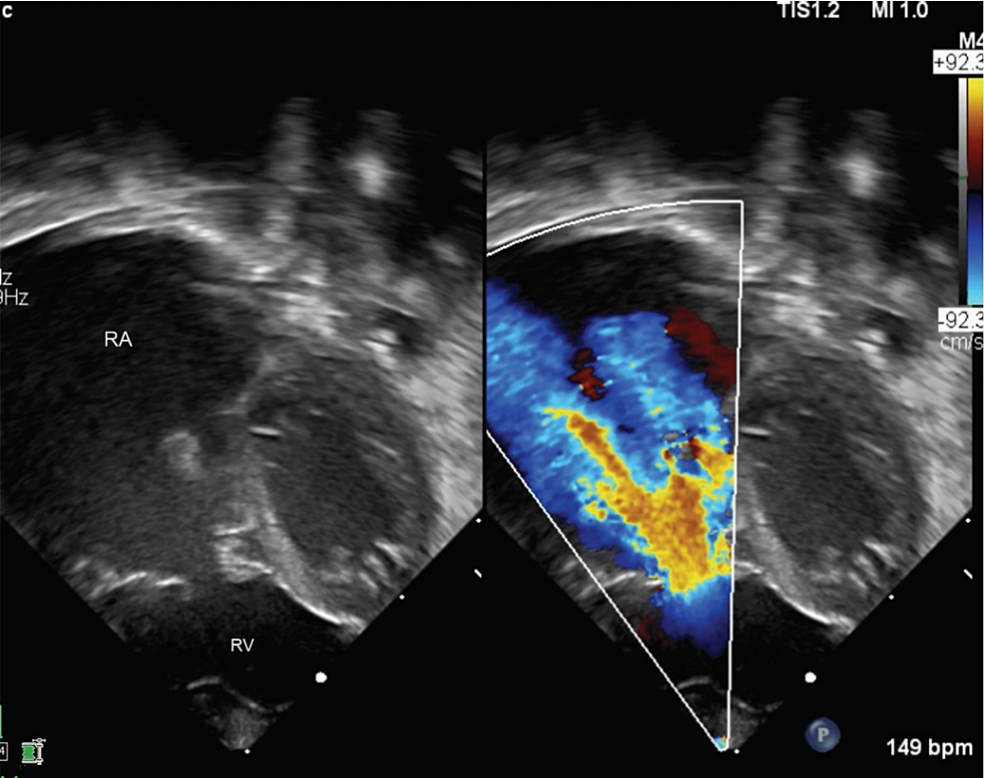
Neonatal echocardiogram showing apical four-chamber view of Twin A with apically displaced tricuspid valve, RA dilation, and severe tricuspid regurgitation RA, right atrium; RV, right ventricle

In the neonatal intensive care unit, an echocardiogram of twin A demonstrated Ebstein’s anomaly with severe TV regurgitation, acquired pulmonary atresia, and marked atrialization of the right ventricle (Figure 3). An enlarged cardiac silhouette was seen on the chest x-ray, consistent with the infant’s known congenital heart defect. Arterial blood gas one hour after birth was remarkable for metabolic acidosis. During the neonatal intensive care unit stay, adequate pulmonary blood flow in twin A was maintained with continuous prostaglandin infusion. Twin A was noted to have a right pelvic kidney on postnatal imaging, rather than right renal agenesis as suspected from prenatal imaging.

Twin B presented to the intensive care unit with hypothermia but was shortly stabilized. An echocardiogram of twin B showed a small patent foramen ovale with left-to-right shunting. The cardiothymic silhouette was normal on the chest x-ray. There was gastroesophageal reflux to the upper third esophagus on the upper gastrointestinal x-ray. Twin B had events of bradycardia and apnea for two consecutive nights that resolved without intervention.

## Discussion

Failure of the Mullerian ducts to fuse during fetal development results in a bicornuate uterus, one of the many congenital uterine anomalies. The American Society of Reproductive Medicine defines a bicornuate uterus as having an external fundal indentation of greater than one centimeter, with the complexity of the uterine cavity and cervix being variable [9]. Patients with bicornuate uteruses can be unicollis or bicollis, have right or left communicating tracts in the uterine cavity, and may also have a uterine septum present. Bicornuate uteruses typically open to a single vagina. Of all the Mullerian malformations, the bicornuate uterus is the third most common [10]. 

Most patients with bicornuate uteruses are not diagnosed until they are trying to conceive or are already pregnant. While some patients may report dysmenorrhea and menorrhagia, having a single vaginal cavity that does not interfere with sexual intercourse or menstruation leads patients not to consider themselves being affected by a bicornuate uterus. Complications in pregnancy include preterm labor, placental abruption, miscarriage, uterine rupture, intrauterine growth restriction (IUGR), and cervical incompetence [10]. Singleton and twin pregnancies have been reported in patients with bicornuate uteruses, and assisted reproductive technology can help achieve pregnancy in those experiencing infertility. Strassman metroplasty has been performed to surgically repair the uterine cavity in patients with bicornuate uteruses [11]. 

This report details a case of a female with a bicornuate uterus pregnant with dichorionic-diamniotic twins, with twin A affected by Ebstein’s anomaly and right pelvic kidney. Ebstein’s anomaly is thought to be idiopathic, but external and genetic factors have been linked to the congenital heart defect. The most well-reported cause of Ebstein’s anomaly is antenatal lithium exposure. The rate of Ebstein’s anomaly in identical twins is higher than in the general population, and mutations in sarcomere protein genes have been identified in those affected by Ebstein’s anomaly [12]. There is also a higher incidence of Ebstein’s anomaly in offspring born to affected women (6%) compared to men (0.6%) [1]. Bicornuate uteruses are known to have extensive obstetrical complications, but the impact on the fetus has been poorly known. While congenital malformations such as omphalocele, limb deficiencies, and anencephaly have been reported in offspring from mothers with bicornuate uteruses, the direct relationship between a bicornuate uterus and congenital cardiac malformations remains unknown [13].

The patient presented in this case did not report any personal or familial history of cardiac defects. Although the patient has a congenital malformation, the currently known genes responsible for the bicornuate uterus and Ebstein’s anomaly are different. Having dizygotic twins, as opposed to identical twins, can explain why twin B was unaffected by Ebstein’s anomaly. Renal and Mullerian anomalies have been linked due to the simultaneous development of the Mullerian ducts and urogenital sinus in utero. Familial inheritance of uterine anomalies has been reported, and the identification of a bicornuate uterus in twin A could explain the pathophysiology behind the right pelvic kidney identified [14]. 

There is no current identifiable cause for twin A having Ebstein’s anomaly as a result of the mother having a bicornuate uterus. As in most patients with Ebstein’s anomaly, we hypothesize this is an idiopathic case. Future evaluation of the uterus of twin A could potentially link the female’s right pelvic kidney to a possible congenital malformation of the uterus. Future identification of signaling factors responsible for mesoderm development can help identify the relationship between congenital malformations that affect both the cardiovascular system and the uterus.

## Conclusions

This case reports a rare dichorionic-diamniotic twin pregnancy in a patient with a bicornuate uterus, with twin A affected by Ebstein’s anomaly and right pelvic kidney. The relationship between the cardiovascular malformation in twin A and the mother’s bicornuate uterus is currently unknown. However, future identification of genes responsible for organs derived from mesoderm can help explain this relationship. Congenital malformations of the uterus have been reported to be familial, and identification of future Mullerian anomalies in twin A can explain the cause of her having a right pelvic kidney. We hypothesize the cause of Ebstein’s anomaly to be idiopathic in twin A, with potential genetic influence on impaired kidney development. It is essential to note the lack of congenital malformations in twin B, which could be explained by the offsprings being dizygotic as opposed to monozygotic.
